# Droplet Detection and Sorting System in Microfluidics: A Review

**DOI:** 10.3390/mi14010103

**Published:** 2022-12-30

**Authors:** Can Huang, Yuqian Jiang, Yuwen Li, Han Zhang

**Affiliations:** 1 Department of Electrical and Computer Engineering, Texas A&M University, College Station, TX 77842, USA; 2 State Key Laboratory of Food Nutrition and Safety, College of Food Science and Engineering, Tianjin University of Science and Technology, Tianjin 300457, China

**Keywords:** droplet microfluidics, droplet detection, droplet sorting

## Abstract

Since being invented, droplet microfluidic technologies have been proven to be perfect tools for high-throughput chemical and biological functional screening applications, and they have been heavily studied and improved through the past two decades. Each droplet can be used as one single bioreactor to compartmentalize a big material or biological population, so millions of droplets can be individually screened based on demand, while the sorting function could extract the droplets of interest to a separate pool from the main droplet library. In this paper, we reviewed droplet detection and active sorting methods that are currently still being widely used for high-through screening applications in microfluidic systems, including the latest updates regarding each technology. We analyze and summarize the merits and drawbacks of each presented technology and conclude, with our perspectives, on future direction of development.

## 1. Introduction

In the past decades, microfluidic systems have been widely used in the fields of drug discovery [1,2,3,4,5,6,7], single-cell studies [8,9,10,11,12,13,14], medical diagnostics [15,16,17,18,19,20,21,22,23,24], and tissue engineering [25,26,27,28,29,30,31,32,33]. Droplet microfluidics, as one of the major branches of microfluidic techniques, exhibits great promise in their ability to conduct high-throughput assays for a broad range of life science applications, including single-cell sequencing, antibody and drug discovery [34,35], cell assays, and pathogen screening [36,37]. To achieve a successful screening, on-the-fly manipulation of droplets plays a key role. Thanks to the extensive technological advancements made over the past decades, individual droplets can now be high-efficiently transported [38,39], merged [40,41], dispersed [42,43], trapped [44,45], and sorted [46,47,48] at very high throughput. Among the variety of droplet manipulation techniques, the droplet sorting technique is considered one of the most critical components during the screening procedure [49]. The goal of droplet sorting is to collect desired droplets for further use. A typical droplet sorter consists of two functional parts: The droplet detection part and the droplet sorting part.

The droplet must be first detected before they are sorted into the collection reservoir. Droplet detection methods would require an active detection system that simultaneously monitors one or multiple indicators from a droplet to determine whether the targets are present. While the indicators can be different types of properties, detectors also vary correspondingly. The selection of detection method relied on the method and limit of detection (LOD) of the biosensing system applied in the droplet, as well as the requirement of screening throughput. Here, we will focus on the three major methods for droplet detection, which are impedance-based detection, fluorescence-based detection, as well as visible light-based detection.

Droplet sorting can be accomplished using pneumatic [50], magnetic [51], thermal [52], acoustic [53], and electric methods [48]. The most common sorting strategy is dielectrophoresis (DEP) where a droplet is deflected by an electric field to exit a device via a selected channel. Other commonly used sorting strategies have been achieved using pneumatic valves and external acoustic waves. Coupling these sorting techniques with rapid analytical assessment can provide high throughput approaches to hit identification and selection, and this enables downstream discovery and analysis.

In this review, we elaborated on the state-of-the-art droplet detection and sorting methods, including the recent advances regarding each technology. We analyzed and summarized the merits and drawbacks of these technologies and concluded with our thoughts on the prospects of development.

## 2. Detection Methods

### 2.1. Impedance-Based Droplet Detection

Electrical impedance spectroscopy (EIS)-based detection utilizes the contrast of impedance between different objects to achieve the detection and has been widely applied in microfluidic systems to characterize particles and cells [54,55,56,57,58]. The typical detection setup contains planar sensing electrode patterns deposited on the glass substrate (sometimes with an inert interstitial layer to isolate electrodes from the solution, thereby reducing electrode polarization) underneath the microfluidic channel [59]. The planar sensing design could have various patterns, varying from being as simple as two parallel electrodes to digital-coded electrode arrays, to achieve different detection functions [60,61,62,63]. Corresponding to the pattern design and sensing mechanisms, an impedance analyzer is required to feed excitation signals to and capture signals back from electrodes. When necessary, a current amplifier can help increase the detectability of signals [64,65].

**Figure 1:**
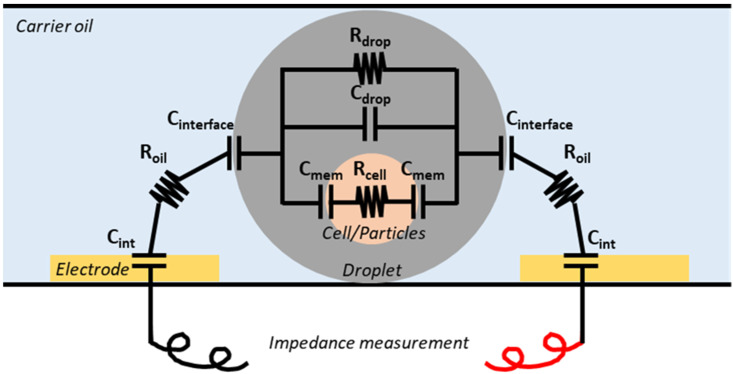
Impedance detection principle. The capacities between oil–water interface (C_interface_), resistance (R_drop_), and capacitance (C_drop_) from droplet solution, as well as the capacitance from the cell membrane (C_mem_) or particle interface, and resistance from cytoplasma (R_cell_) or the particle solid content result in the detectable impedance changes that can help with the determination of droplet contents.

In the case of droplet microfluidics, the circuit model of the blank microchannel in a carrier oil, the empty droplet, or the droplet-containing cells/particles are all different due to their intrinsic impedance characteristics (Figure 1). When the droplet is present in the detection system, additional capacitance from the oil–aqueous interface, as well as capacitance and resistance from the droplet (or plug) itself, will be introduced into the sensing region [66]. Additionally, if the cell/particle is present inside the droplet, its cytoplasm or solid content, cellular membrane, and inner membrane (if any) would all introduce additional impedance compartments in the equivalent circuit models and result in impedance changes. Based on this detection principle, several applications have been developed to achieve droplet characterization and in-droplet content evaluation. Gu et al. developed an electrochemical sensor for highly sensitive glucose sensing [67] by evaluating the enzyme activity assay in flowing droplets. Yakdi et al. introduce the sensing methods that can be used to characterize the droplet size and speed based on impedance fluctuation [68]. With regard to in-droplet content evaluation, Kemna first developed an analytical method to distinguish viable and non-viable cells, as well as methods to acquire information such as the size, number, and morphology of in-droplet cells [69]. Marcali et al. first achieved hemagglutination detection using impedimetric sensing [70]. Fan et al. studied the osteogenic differentiation of single cells that encapsulated inside droplets to undermine the correlation between membrane capacity and the appearance of two biomarkers [71]. Cao et al. also utilized impedance-based detection to evaluate the bacterial growth and to assess the dose response of two soil bacteria [72]. This handful of applications has demonstrated the flexibility of impedance-based detection methods when dealing with various types of biological samples for screening purposes.

Overall, impedance-based sensing requires only basic fabrication and handling skills. However, due to the highly localized nature of the sensing region provided by planar electrodes, the accuracy and sensitivity are non-trivial to be optimized. Impedance sensing often requires shallow sensing channels with narrow electrode gaps [73], which might introduce unwanted channel fluidic resistances that hurdle microfluidic operation. This issue might be able to be resolved by fabricating microchannels with variable heights with an advanced photolithography method to only decrease the channel height at the detection region [38,74]. Additionally, to provide better impedance contrast to enhance detection, different frequencies might be required to fit application scenarios [8,64], and sometimes, low conductivity media are also essential to facilitate the detection by reducing the background signal from aqueous media [69]. Some level of expertise would be preferred to obtain the best readings. Lastly, impedance measurement provides limited distinguishing power against cells with similar physical properties, e.g., different types of bacterial cells, two different mammalian cells, or a heterogeneous subspecies population, which increase the challenge for themselves to be broadly applied against functional screening.

### 2.2. Fluorescent Emission-Based Droplet Detection

Fluorescent emission-based detection is commonly used in microfluidic systems for droplet detection due to its low background, high sensitivity, and fast response time [75,76,77]. So far, fluorescence assays have been utilized for various applications such as directed evolution of enzymes [78,79], single-cell analysis [80], antibody screening [81], and digital PCR [82]. Additionally, the FACS (Fluorescence-Activated Cell Sorting) system has been developed to help biological analysis on the cellular level. In a fluorescence-based droplet detection system, the droplet-encapsulated fluorescent molecule (either introduced by autofluorescence from cells [83] or straining dye [84,85,86]) is first excited from the ground electronic state to the vibrational state in the excited electronic state by absorbing a photon. Then, the excited molecule loses vibrational energy and drops down to one of the various vibrational levels of the ground electronic state by emitting a photon [87,88]. The emitted photons have different energies and frequencies; thus, the structures and characteristics of molecules can be determined by analyzing the different frequencies of the light emission. Typically, the fluorescence intensity at certain frequencies is used as the detection metric; however, the fluorescence lifetime can also be applied to distinguish two different fluorescent compounds on the basis of the corresponding fluorescence lifetime [89,90].

**Figure 2:**
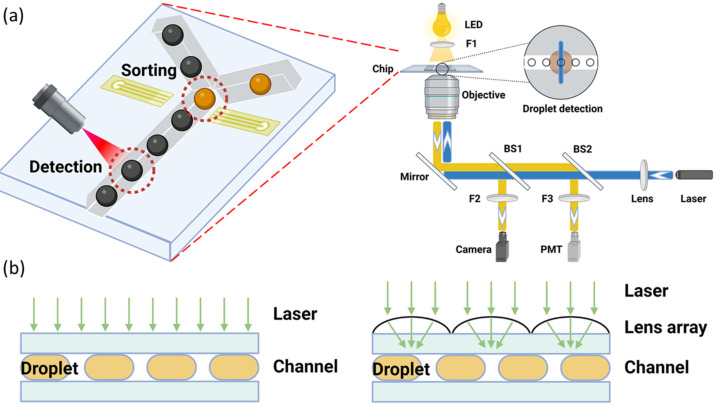
(**a**) Fluorescence detection sketch. L1, L2: focus lens; F1, F2, F3: filters; DM1, DM2: dichroic mirrors; BS1, BS2: beam splitter. Laser light passes through a dichroic mirror focus lens and beam splitters then focus on the microfluidic sample droplet, which conducts the fluorescence staining in the droplet and emits light. The emission light signal was received by the PMT passing through the dichroic mirrors beam splitter and filter. The band passes filter F1 and is used to block the background light wavelengths. The red LED on the top with a red filter 1 is used for the bright files. The real-time images were captured by the camera through a 10× objective. The notch filter 2 is used to block the light, which has a wavelength the same as the laser, to protect the camera. (**b**) Comparison of conventional droplet-based microfluidic device with the micro-optical lens array device. Left: The conventional device. The fluorescence signal is limited on spatial resolution due to signal overlapping. Right: system with the micro-optical lens array, which can improve the excitation light intensity and fluorescent signal collection.

Typical one-color detection stations require light manipulation schemes to introduce excitation light from free space lasers through the microscope to focus the laser on the passing droplets [91]. Figure 2a shows the sketch of the fluorescence detection system in droplet microfluid. The droplets continuously flow through an excitation laser as laser light passes through a dichroic mirror and focuses on the droplet sample. Mercury lamps or LEDs can also be used as excitation light sources. However, the laser has high energy power, as well as a smaller focal spot and beam width, which can provide better sensitivity and limit of detection, especially for faint florescent cases. Lasers can further reduce the minimum exposure time for droplet detection and, hence, increase the throughput. After fluorescence is emitted from the droplet, the emitted light is filtered so that the photomultiplier tube (PMT) could receive desired wavelength light and send a positive signal to the following sorting system. PMT can achieve fast and high performance in signal normalization compared with other signal detection methods such as fluorescent cameras. To support the simultaneous detection of multiple bio-samples with a wide range of fluorescence excitation and emission spectra [92], multicolor fluorescence detection, which configured with multiple color channels and filters, is essential for a variety of droplet-based microfluidic screening applications [93,94]. By applying the optical fibers to combine the multiple-color laser channel [95], the multicolor fluorescence detection system can be made simpler and more compact while allowing all emitted light to be captured with one photodetector [96]. A field-programmable gate array (FPGA), which performs measurements with a resolution in the order of tenths of picoseconds, can be applied to carry out fast fluorescence diagnostic measurements [97,98] to accelerate the signal processing rate in the high-throughput detection system.

Signal detection is usually done by PMT, photodiodes, or imaging processing generated by fluorescent cameras. These conventional detection systems are limited on massively parallel detection in droplet microfluid. There is a conflict between the field of view and optical sensitivity. For the optical setup, a lens with a large numerical aperture will be able to visualize finer details than a lens with smaller numerical aperture. It can help to collect lighter and provide a brighter image for detection sensitivity. Large numerical apertures, such as the single-focal lens, coupled with the PMT can offer high sensitivity, but they are limited to a small field of view for multi-point measurement. The small numerical aperture objectives with a large field of view limit low optical sensitivity. Applying the micro-optical lens array for droplet-based fluorescence is another optical method to increase the fluorescence signal and improve the spatial resolution [99,100]. The schematic of the device is shown in Figure 2b. The integration of micro lenses and mirror surfaces in the detection system can enhance imaging resolution and sensitivity. In addition, a mirror structure on the channel wall induces optical resonance to enhance signals. As a result of keeping the field of view in the overall optical system, the fluorescent signal intensity can be improved.

Overall, fluorescent detection relies on the fluorescent intensity of a droplet. However, the stability and intensity of the fluorescent signal is highly related to the system response time, the droplet throughput, and the method of data collection. In addition, photobleaching and biocompatibility, generally, would raise some concerns that affect the accuracy of the fluorescence detection. Some light-sensitive samples may lose pigment, such as chlorophyll or rhodopsin, when illuminated [101]. Some biological samples would cause irritation, damage, or toxicity under light illumination, which will lead to biocompatibility issues [102]. The cost of the entire setup is also much higher than the impedance detection method. With all being said, the fluorescent detection method is still one of the most used methods due to its flexibility, high throughput, and precise nature.

### 2.3. Other Visible Light-Based Droplet Detection

**Figure 3:**
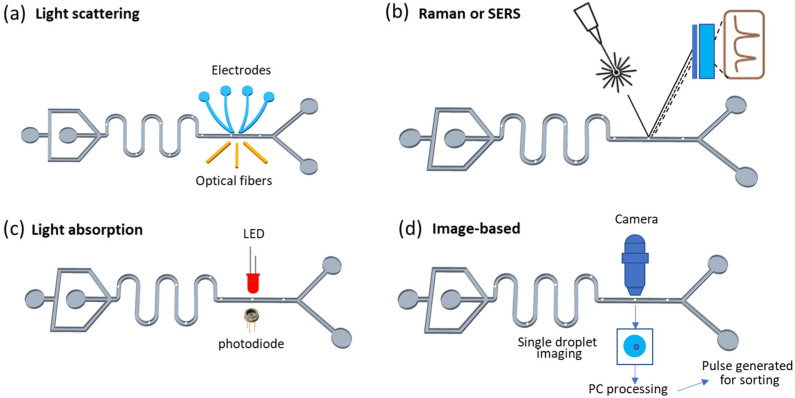
Schematic illustration of droplet detection approaches based on visible light. (**a**) Light scattering-based method integrated with multiple optical fibers; (**b**) Raman or surface-enhanced Raman scattering method for droplet detection; (**c**) light absorption-based method integrated with LED-photodiode pair; (**d**) image-based droplet detection coupled with rapid high-resolution camera.

The emission-based droplet detection methods have been introduced in Section 2.2, and other visible light-based droplet detection approaches will be discussed here, including light scattering, Raman scattering, absorbance, and imaging, which have also been developed [103,104,105,106]. This type of detection obtains information on droplets or inside analytes from reflected light color change, incident light scattering, light absorbance, and image capturing and analysis. Compared with fluorescence-based methods, the most obvious limitation of light scattering approaches is the detection sensitivity. Therefore, appropriate optical fibers (working for light guiding or acting as target sensing after modifications) and cameras with high speed and resolution are generally required for light guiding to transmit and collect signals from droplets. Light scattering occurred when droplets encapsulated with cells or analytes—or empty droplets with solvent alone—are illuminated with incident light (Figure 3a). The scattering light is deflected in different directions, and the distinct distribution pattern will be collected by single or multiple optical fibers positioned at different angles, which can be processed to reflect the size, shape, density, uniformity, and even chemical compositions related to the droplets and inner contents [107]. As a cell detection technique, light scattering has been widely used in flow cytometers for cell analysis and sorting [108]. However, the sensitivity of light scattering-based detection is relatively low, as it is mostly for qualitative analysis in droplets and, typically, not meant for high specificity characterization or functional screening applications.

Compared with visible light scattering, Raman scattering is much more powerful and can even specifically distinguish multiple chemical compositions simultaneously in a complicated solution [109,110,111,112,113,114]. Surface-enhanced Raman scattering (SERS) improved the detection sensitivity as the analytes are approaching or adsorbing metal substrates [115,116]. SERS overcomes the limitations of Raman scattering, such as long acquisition time, requirement of fixed samples, or presence in laminar flow [117,118]. Labeled and label-free detection based on SERS has been developed for sorting microfluidic droplets in-flow [119,120]. The label-free SERS detection is suitable for those targets with strong Raman signals. For labeled SERS-based droplet detection, immuno-modification is used to analyze certain targets [121]. Figure 3b shows schematic Raman scattering and SERS-based droplet detection. Raman scattering technologies require relatively long response times and, therefore, have limited throughput; additionally, the cost of setup is also typically high.

The absorbance of target-containing droplets has also been used as label-free detection signals which, therefore, can trigger the sorting of droplets (Figure 3c). Limited to optical path length, the sensitivity of absorbance measurements can be improved by the elongation of the channel, extension of residence time, or integration of other techniques such as fluorescence-based and UV–vis spectra-based techniques [122,123] Future development may extend to full absorbance spectra and integrate analysis by artificial intelligence [124,125]. Due to the advances in cameras, processing hardware, and machine learning software with image processing algorithms, bright field images of droplets have been able to be real-time captured and processed into signals for droplet sorting (Figure 3d) [126]. Image-based detection overcomes the limitation of flow cytometers in distinguishing objects with similar sizes and morphologies [127]. To enhance the accuracy and efficiency of detection, deep learning algorithms have been increasingly used to classify images for further droplet sorting and selection [125]. Overall, the cost and throughput of absorbance detection methods depend on the choices of optical detection instrumentation, which vary vastly. Nevertheless, the specificity of this method remains fairly low.

## 3. Droplet Sorting Methods

Droplet sorting methods are usually composed of a signal feed to initiate actuators (typically from detection systems), as well as an on-demand actuator system that can manipulate one or a few droplets to be deflected from a droplet train to a separate collection. So far, several passive methods have been developed for droplet sorting, namely deterministic lateral displacement (DLD) [128], microfiltration [129], and inertial separation methods [130]. These technologies achieve sorting of target droplets by utilizing their physical dimensions, and they can potentially simplify the screening system by skipping the detection unit when the biological models actually affect the droplet sizes [131]. For example, growth-associated droplet shrinkage phenomena can be used to sort droplets encapsulating the proliferated cells from a single-cell encapsulation library, as well as achieve bacterial quantification and growth monitoring [132]. However, for most of the droplet screening assay, the encapsulated cells and their biological responses would not affect the size of the droplet during the entire biological interrogation therefore, having solely passive sorting methods without a forehead detection unit would not be applicable for screening scenarios when droplets with or without cells of interest share similar dimensions or mechanical properties. In this section, we focus on the active droplet sorting functions, which can selectively manipulate target droplets from other droplets with the same physical properties. Pneumatic, dielectrophoretic, and acoustophoretic, as well as other common sorting methods, are discussed in detail. The typical droplet sizes employed for these active sorting methods range from tens of microns to hundreds of microns [49].

### 3.1. Pneumatic-Based Droplet Sorting

Pneumatic-based droplet sorting was one of the earliest developed droplet sorting technologies. It utilizes the air pressure to deform the elastic PDMS microchannels or valves, achieving sorting by introducing either disturbance of flow or physical blockage of the downstream channel [50,133,134,135]. Here, the PDMS elasticity is critical, as the mechanical deformation of PDMS under pressure determines the effectiveness of the sorting function. The following section reviews all types of the PDMS-related, pneumatic-based droplet sorting technologies that have been developed so far.

**Figure 4:**
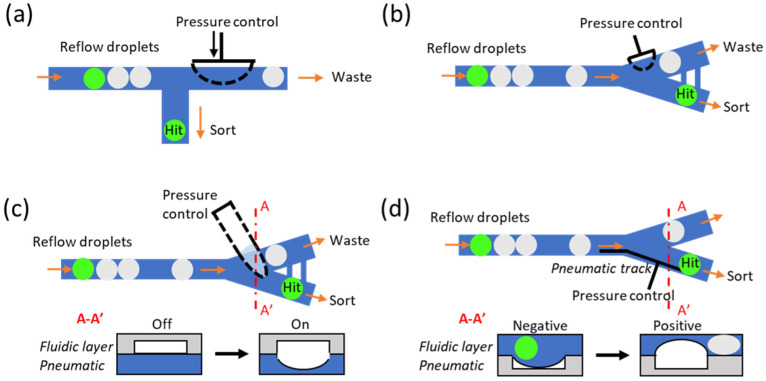
Variety of different versions of pneumatic based droplet sorters. (**a**) Very first pneumatic sorter that uses a side channel to block the main downstream channel to deflect hit droplets into the bottom channel; (**b**) sorter with a valve that directly points towards the tip of the bifurcation structure and creates subtle disturbance to deflect hit droplets to the opposite channel; (**c**) bilayer structure that deforms the ceiling of the fluidic channel to achieve physical blockage, and A–A’ shows the cross sectional structure before/after the pressure is applied; (**d**) pneumatic rail design (black line) that can achieve guiding (negative pressure) and blockage (positive pressure) functions, and A–A’ shows the cross sectional structure before/after the pressure is applied.

Both single-layer valves and multi-layer valves have been implemented into the microfluidic system to perform the droplet sorting function. In the case of single-layer valves, Abate et al. first proposed the single-layer valve system which positions an adjacent pneumatic channel next to the main streaming channel to achieve the droplet sorting function by actuating the thin sandwich membrane with positive pressure (Figure 4a). The positive pressure would extrude the thin membrane into the main channel to obstruct the fluidic flow and detour the upcoming droplets to another hit chamber [136]. Later, Abate et al. improved the system by simplifying the sorting design into a bifurcation structure and introducing the shunt structure to balance the fluidic resistance to minimize the sudden pressure fluctuation, as well as replace the adjacent channel to a valve that directly points towards the tip of the Y junction [137]. Due to the calibrated asymmetric design of the microchannel, the mainstream droplet would go directly to the “waste” channel if the valve is not actuated. When the valve is actuated, the fluidic resistivity dynamic would be slightly changed so that the target droplet would be guided toward the opposite channel as the “hit” droplet (Figure 4b). In the case of multi-layer valves, with a similar concept aiming to disturb the fluidic dynamic by sudden channel deformation, Jin et al. introduced another method to achieve droplet sorting in the format of a bilayer valve [138]. The pneumatic valve is aligned and placed on the top of the fluidic channel; therefore, it can improve the sorting performance and reduce the minimal valve actuation time (Figure 4c). Zhang et al. introduced bilayer dynamic pneumatic rails to enable droplet sorting by adjusting the pressure balance between oil and the pneumatic channel [139], presentting good accuracy but fairly low throughput (Figure 4d).

Overall, pneumatic-based droplet sorting systems are relatively easy to fabricate and operate with simple instrumentation. Such types of systems are cost-effective and compatible with most biological systems because they do not introduce any electrical, acoustical, or thermal disturbance on biological samples inside droplets. However, because each sorting event is based on the mechanical deformation of the PDMS layer, it has very limited throughput compared with acoustic and electrical methods. Additionally, the hydrodynamic balancing of the entire system would require certain expertise and carefulness to ensure the proper sorting/skipping functions can be realized against different droplet sizes/flow profiles [140,141]. Moreover, the robustness of the pneumatic devices is also a major concern, especially when running for a long-term screening task. Overall, in the case of high-throughput droplet microfluidic assay, it has been mostly replaced by dielectrophoretic methods and acoustophoretic methods to match the throughput of other components from the droplet system.

### 3.2. Dielectrophoretic-Based Droplet Sorting

Among various sorting methods, the DEP method is the most widely used method due to its advantages of robustness, high accuracy, and selectivity, as well as high sorting rates/response times. The DEP method utilizes the dielectrophoretic response from an aqueous droplet to generate a positive DEP force (meaning the force with direction pointing along with the increasing electric field) on the entirety of the droplet to displace the droplet from their original trajectory to achieve sorting. In general, the DEP sorter can process up to 100 million samples per hour [142], and it allows the sorting of a large range of droplet volumes from 20 fL to 10 nL [143,144]. Compared to state-of-the-art robotic screening systems, the DEP-based droplet platform performs the entire assay with a 1000-fold increase in speed and a 1-million-fold reduction in cost [142,145,146,147]. The DEP methods can be classified into direct current (DC) [148,149,150,151,152] or alternating current (AC) [153,154,155,156] methods. However, compared with the AC method, the DC method is rarely implemented in recent years due to the fact that DC is less controllable, has low stability at high voltage, and is more difficult to amplify compared with AC.

#### 3.2.1. Early Version of DEP Sorters

**Figure 5:**
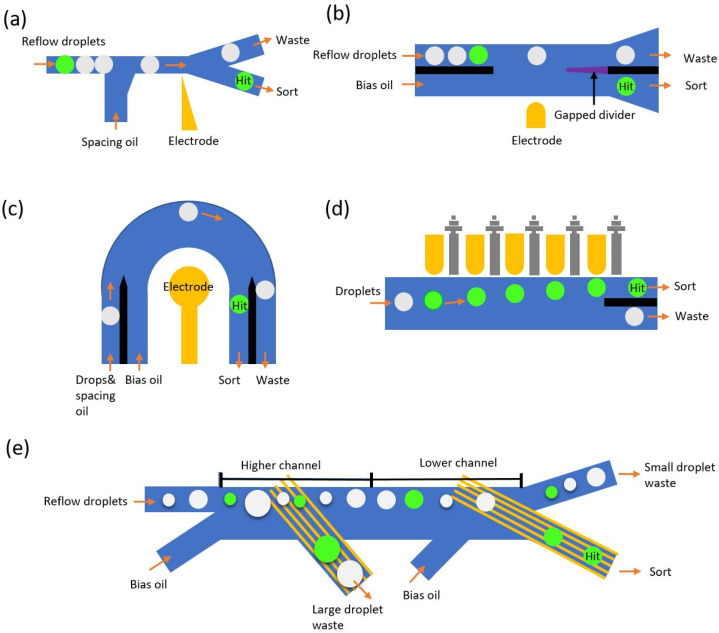
Variety of different types of DEP droplet sorters. (**a**) A typical first-generation DEP droplet sorter; droplets with color dye are sorted by DEP force into the bottom channel. (**b**) A schematic of the fast droplet sorter, with detected and selectively displaced green droplets being separated by a gapped divider (purple) of reduced channel height. (**c**) Schematic of the concentric design with inlet and outlet channels labeled. Black areas represent oil bypass regions generated by double-layer fabrication. (**d**) Schematic of the SADA-based fluorescence-activated droplet sorter (SADA sorter in short). (**e**) The working principle and design of the size-based droplet manipulation scheme using IDE-generated DEP force and the droplet buoyancy phenomena. This top view of the droplet manipulation region illustrates that larger droplets will follow the trajectory of the surface IDE patterns, while the smaller droplets will continue to flow to the waste outlet uninterrupted by the IDE-generated DEP force.

A typical first generation of DEP sorter consists of a Y-shape sorting junction linked by two-way channels. There are two electrodes placed near the fluidic channel (positive electrode and ground electrode). When there is no electric field, droplets flow into the branch with lower resistance (the collection channel normally has higher resistance than that of the waste channel). Otherwise, an electric field is triggered by the detection part, and DEP forces steer the droplets into the collecting channel (Figure 5a) [77,78].

Although it provides many advantages, the early DEP sorter also exhibits some drawbacks that can affect its performance. Firstly, the hydrodynamic resistance balancing that guides negative droplets to the waste channel can be unstable, leading to a failure of collection and drop splitting at the sorter exit junction due to the change of flow rate, droplet size distribution, and the number of droplets presenting in the system. As a result of this, the false positive and false negative ratio may increase [48,157]. Secondly, conventional linear geometries of DEP electrodes use a straight channel with the sorting electrode positioned at a point down its length; this produces weak DEP forces up and downstream but strong ones directly opposite the electrode. Most of the drop deflection occurs over the short time the droplet is directly in front of the electrode. Such a design limits the effective sorting region in the device and, therefore, requires a much larger applied voltage in case of screening at high throughput. However, increasing the voltage to above 1800 Vpp (Peak-to-peak Voltage) could cause tip streaming of the droplets, resulting in depolarization and a much lower sorting efficiency [77,78].

#### 3.2.2. Improved DEP Sorters

Conventional planar electrodes that are used for DEP sorter in early versions are replaced by using one-step fabricated three-dimensional (3D) metal electrodes [158,159,160]. The electrodes are fabricated by injecting liquid metal into the microchannel. Compared to the 2D counterparts, 3D electrodes could generate a stronger and more homogeneous electric field. It helps increase the deflecting velocity and reduces both the response and the recovery times for droplets deflecting and returning to the center of the channel, respectively. Furthermore, the voltage needed to generate the electric field with 3D electrodes can be significantly reduced compared to the 2D planar electrodes. The electrodes also don’t require alignment, as the microchannel filled with liquid metal is fabricated using the same lithography process.

Instead of controlling the waste droplet steam using the hydrodynamic resistance method, Sciambi et al. [142,146] developed an ultra-high-speed droplet sorter that utilized a spacing oil flow bias of the droplets to the channel side away from the electrode (Figure 5b). The energized DEP electrode then attracts the droplet while triggered. The droplets then move downstream to a longer divider with part of it squeezed by the gap. Due to this change, the small lateral displacement by DEP will increase—since the droplet travels downstream—and force the droplet into the hit channel, while the non-affected droplets will remain on the other side. Since the first displacement by the electrode is small, the operating time needed for one droplet is relatively short. The first improvement of this design is the addition of oil bias flow. The initial lateral displacement provided by the bias oil flow could improve the rejection of false positives and false negatives, which are caused by imbalanced hydrodynamic resistance. Besides, they introduced a soft-gapped divider, and this divider could help to reduce the droplet splitting and tip streaming issue [142] while screening at a high flow rate. This results in an ultrafast sorting speed with the highest rate ever reported of approximately 30 kHz. Although most droplets could be correctly sorted, certain errors cannot be neglected. For example, uniform spacing is crucial to the sorting process. High flow speed would cause the droplet to stack vertically in multiple layers and lead to irregular spacing and droplet size change. Reflowing droplets with an abnormally large or small size could also fail in sorting due to the non-uniformed droplet spacing caused by size change. One possible way to improve the sorting accuracy could be the removal of the incorrect-sized droplets by a filtration method before the sorting procedure [128,130,161,162,163,164,165,166].

Another sorting device introduced by this group made use of a new sorting geometry that uses concentric electrodes to increase the time over which the DEP force acts and, thus, the larger displacements that can be achieved at equivalent speed and voltage conditions (Figure 5c). Different to the conventional linear sorter that provides a gradient electrical field, the new concentric geometry provides a uniform electrical field across the arc of the sorting junction. This design allows the target droplets to experience the same DEP force from the beginning to the end of sorting. Their results show the concentric sorter was able to sort droplets at the same flow conditions with three times fewer applied voltages. This allows the sorter to operate while causing less tip streaming of droplets.

During the droplet sorting process, as the throughput increases, larger droplets break into smaller pieces more easily than smaller droplets due to the increase in shear stress and voltage applied. To ensure the structural integrity of the droplets and, thus, the reliability of the results and reproducibility of an experiment, Isozaki et al. [160] present a sequentially addressable dielectrophoretic array (SADA) chip that overcomes the aforementioned trade-off, enabling high-throughput sorting of large droplets without any damage to the droplets (Figure 5d). Instead of using one strong electrode, they used an array of electrodes that gently pulled the target droplet multiple times to the predesigned channel. The sorting efficiency can reach as high as 98.6% for 140 pL droplets. They also demonstrated that the SADA chip can sort droplets to more than two outlets. This achievement creates the possibility of sorting samples that consist of multiple populations and rare targets [167]. However, since only one laser sensing/feedback control is utilized in front of the first electrode in the array, the tolerance to polydispersity of this SADA can be problematic since the flow speed of a droplet, under a certain operation condition, is size-dependent.

In most applications, highly monodispersed droplets are required, as each of these functions is designed and optimized for operation with droplets of a specific size range. If input droplet size varies or falls outside of the optimized size range, then high error rates in the droplet manipulation steps occur. The size change of droplets commonly occurs during droplet incubation, transition, and other manipulation procedures. This outcome, in turn, leads to lower performance of the entire droplet microfluidics platform. The current state-of-art droplet sorters are designed to perform at certain droplet sizes, and the tolerance to polydispersity is low. To improve the sorter performance in the case of polydispersed input droplets, Dr. Han’s group has developed an interdigitated electrode (IDE)-based droplet sorter (Figure 5e) [168]. The system uses a highly localized electric field, generated at the bottom of the microfluidic channel using IDEs and coupled with the fact that droplets float up due to their natural buoyancy in a carrier oil, to selectively manipulate only droplets in the desired size range. By sequentially linking the high-pass and low-pass sorter designs, selecting only a certain droplet size range becomes possible, with a filtration efficiency of more than 99%, at throughputs of up to 100 droplets/s. It can be expected that the combination of a fluorescent detector with the IDE size-based sorter can easily achieve a two-metric sorter that sorts droplets according to their fluorescent signal and size.

In conclusion, a dielectrophoretic-based droplet manipulation device is fairly easy to fabricate with the highest accuracy and throughput reported, and it can be applied against most of the biological and non-biological samples. It is definitely one of the most practical and robust systems to be used in active sorting applications.

### 3.3. Acoustic-Based Droplet Sorting

Acoustic-based droplet manipulation is another method that can achieve high throughput droplet sorting functions. Generally, acoustic waves are generated by piezoelectric actuators and prorogated through bulk (bulk acoustic wave, BAW) or surface (surface acoustic wave, SAW) materials, across the droplet streaming channel, to induce an acoustophoretic response on passing droplets to achieve sorting functions.

**Figure 6:**
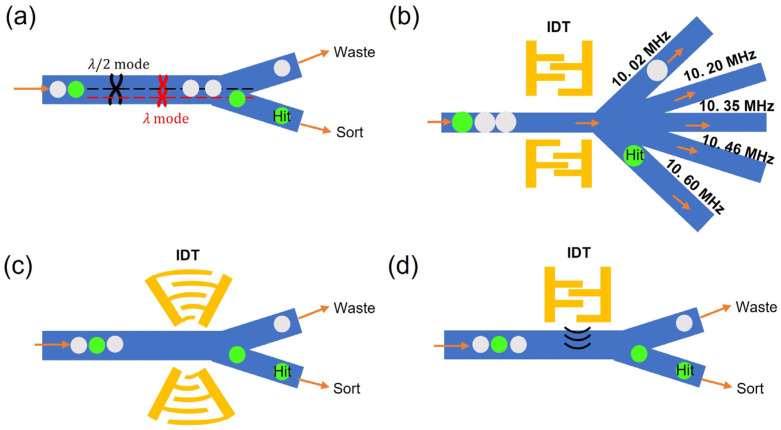
Variety of different versions of acoustic-based droplet sorters. (**a**) BAW droplet sorter that utilizes the switching of λ and λ/2 mode to achieve the droplet sorting towards different downstream channels. (**b**) A SSAW droplet sorter that can utilize two pairs of standard IDTs to sort droplets toward different downstream channels by using different actuation frequencies. (**c**) Developed single-phased, focused transducer method that utilized a revised IDT structure to achieve sorting with higher energy efficiency. (**d**) TSAW droplet sorter that pushes all droplets towards the bottom channel when switched on.

BAW methods require the use of materials with high acoustic impedance (such as silicon or glass) as the main fluidic channel. Typically, the acoustic transducer would be physically attached underneath the bulk materials to generate the propagation acoustic waves affecting the droplets, and the channel width has to strictly comply with the equation w = C_f_ n/(2f_res_) (here, w is the width of the microfluidic channel, C_f_ represents the speed of sound in the surrounding fluid, *n* = 1, 2, 3 represents first, second, third harmonic, f_res_ is the resonance frequency of the piezoelectric actuator used) in order to generate a standing acoustic field with stable acoustic nodes for manipulation. When droplets are subject to the acoustic field, because of its positive acoustic contrast factor, droplets will preferentially migrate towards the pressure node and, therefore, achieve the selective sorting function [169]. Failing to pair the piezoelectric transducer with the channel dimension will result in a decrease in manipulation efficiency and severe heat generation. Therefore, BAW, typically, would require deep reactive-ion etching (DRIE) to fabricate the microfluidic channel with the precise dimensions that are desired. Leibacher et al. have demonstrated a droplet sorting system using BAW where the droplets can be actuated by a generated standing acoustic field and migrate to the pressure node due to its positive acoustic contrast factor (Figure 6a). As they changed the excitation frequency from full lambda to half lambda mode, the nodal position shifted between the upper and lower channel, resulting in sorting functions on droplets [170].

SAW methods utilize acoustic wave propagation through the surface of the piezoelectric substrate to manipulate droplets [171,172,173]. Lithium niobate (LiNbO_3_) is with excellent electro-optic and piezoelectric properties [174]; thus, it is often used in this application as the substrate. Interdigitated electrode transduces (IDT) are commonly used to generate the SAW, where the dimensions of the interdigitated electrodes will determine the resonance frequency [175], and subsequently, the manipulation efficiency, as the higher resonance frequency can deliver higher induced acoustic body force [176,177]. Compared with BAW, SAW has fairly easy fabrication complexity, as it can be coupled with PDMS microchannels, and it only requires a standard metal deposition and patterning fabrication process to deposit IDT on the piezoelectric substrate [178]. In terms of the manipulation methods, SAW has been utilized with two different modes to achieve active droplet sorting.

With the first method, similar to the BAW manipulation method, SAW can also generate the standing acoustic field (SSAW) to create a stable acoustic gradient by having two IDTs countering SAW at the same resonance frequency. Li et al., in 2013, demonstrated the droplet sorter with this SSAW method to achieve selective multichannel droplet sorting, utilizing IDTs at various resonance frequencies (Figure 6b) [179]. Further, Zhong et al. have developed a single-phased, focused transducer method with improved energy transmissions and achieved throughput higher than 1000 droplets/s with less than 20 V_pp_ input signal (Figure 6c) [47]. Such development opens the SSAW sorting techniques with subsequent droplet-based single-cell cultivation and analysis applications.

Additionally, traveling acoustic wave (TSAW) methods have also been demonstrated for droplet sorting applications. TSAW only requires one single pair of IDTs to generate the surface acoustic wave. When the propagated surface acoustic wave passes underneath the microfluidic channel, the mismatch of the speed of sound will result in an acoustic radiation effect [180] that induces acoustic body force along with the radiation direction. When droplets are subject to this TSAW, droplets receive such acoustic force and migrate toward the radiation direction. Franke et al. first demonstrated the use of TSAW to sort droplets with this concept (Figure 6d). A bifurcation design was used where the branch that is closer to the IDT actuator has been designed to be with lower flow resistance. When the TSAW is induced, the deflected radiation acoustic wave acting on the passing droplet pushed all droplets into the opposite branch [181]. This method has been utilized and demonstrated with various sorting applications [182,183].

With the high throughput nature, acoustophoretic methods and dielectrophoretic methods have become the two most promising types of methods to achieve high throughput functional droplet screening. However, acoustophoretic methods also have certain limitations. First, the acoustic force is determined by the compressibility of the carrier oil and aqueous medium, as well as the size of the droplet, applied frequencies, and matches between actuators with a microfluidic chip. With these many affecting factors, certain expertise and consideration are required to be able to design a functional sorting system. Additionally, the acoustophoretic method often encounters energy efficiency issues. For example, because the maximum size of droplets that can be manipulated by SSAW is limited with ¼ of a standing acoustic wave, lower frequency has, therefore, been commonly used to enable manipulation with the larger droplet. However, this is done at the cost of less energy delivery efficiency. TSAW has less spatial and energy confinement due to its scattering nature. In the case of BAW, a slight mismatch between the actuator and microfluidic dimensions will cause severe heat dissipation issues. This might induce biocompatibility concerns if not resolved properly. Finally, the cost of such a manipulation system is comparably high because of the usage of a LiNbO_3_ substrate or a DRIE fabrication process. Overall, we think DEP methods are still favored in most cases.

### 3.4. Other Active Sorting Methods

Aside from the droplet sorting methods described above, magnetic-based [51,184,185,186] and thermal-based droplet sorting [52,187,188] can also be utilized in droplet microfluidic systems. Certain applications have been reported utilizing these two techniques in the past; however, they have shown a tendency to be deployed less due to some inevitable technical constraints.

**Figure 7:**
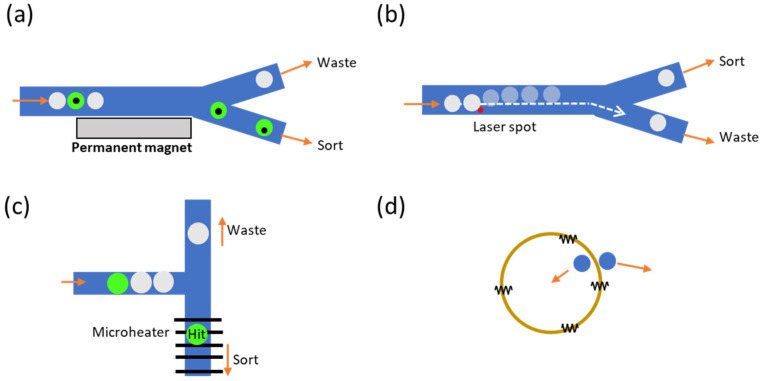
Variety of different versions of other droplet sorters. (**a**) A magnetic droplet sorter that can utilize either permanent magnet or electromagnet to achieve selective droplets with magnetic beads labeled droplets. (**b**) A laser beam focused spot can generate a localized thermocapillary effect, so that force can be generated at the oil–aqueous interface, to deter the trajectory of droplets. (**c**) Resistive heating at the downstream channel would decrease the fluidic resistance and achieve droplet sorting function. (**d**) An optical tweezer that can manipulate the droplets near the laser ring.

In terms of magnetic-based droplet manipulation, there are, essentially, two key components: external magnetic field and affecting magnetic materials. External magnetic field can be induced by an electromagnet or a permanent magnet. Electromagnet field introduces on-demand control in terms of intensity and on/off magnetic field. However, it increases the complexity of the system and introduces unwanted heat dissipation. Permanent magnets, on the other hand, can only have limited control over generated magnetic field and, therefore, can be only used when intrinsic magnetic properties of droplets can be used as sorting criteria (Figure 7a). In terms of magnetic materials, ferrofluidic and magnetic beads are often used to induce magnetic force in order to achieve effective sorting. This introduced additional labeling steps before sorting or, sometimes, even biocompatibility issues when it comes to biological targets. Additionally, compared with dielectrophoretic methods, although with fairly comparable fabrication and operation complexity, magnetic manipulation has very limited throughput—typically up to 10 droplet/sec—which would not be very useful in the high-throughput screening scenario that droplet microfluidics are often applied for. In conclusion, magnetic-based sorting has quite limited applications, as dielectrophoretic methods and acoustophoretic methods can cover most other scenarios with better overall performance.

Heating elements in droplet microfluidic systems can also be utilized as thermal-based droplet sorting methods in certain cases. Laser beam-focused points can also create localized thermocapillary effects, so that force can be generated at the oil–aqueous interface, to deter the trajectory of droplets (Figure 7b); resistive heating can be utilized to achieve droplet sorting at 0.5 droplet/sec throughput and with a potential chance to induce droplet splitting (Figure 7c). Such manipulation techniques have been reported both as a fix-point sorting method to provide on-demand sorting for sequenced droplets, as well as acting as an optical tweezer to enable 2-D manipulation on certain droplets to achieve moving/merging functions (Figure 7d) [189]. Nevertheless, as the throughputs reported are low and the system requires excessive calibrations in order to guarantee sorting accuracy, very limited applications would find thermal-based droplet sorting methods to be the best option.

## 4. Discussion

In this review, we summarized all recently developed technologies in microfluidic droplet detection and sorting. All the presented technologies have been validated against real biological assays; however, due to the nature of the system setups and requirements, they might ideally fit in different scenarios.

For droplet detection, in the case of high-throughput droplet screening applications, three types of droplets are typically examined at the detection phase: empty droplets, droplets containing cells of interest, and droplets containing unwanted cells. Knowing that droplets containing cells of interest would only consist of a tiny portion of the entire droplet population, having decent throughput while ensuring high distinguishing power becomes the most critical performance metric. We have summarized the analytical parameters for the aforementioned detection methods, including their detection throughput, system cost, and reported applications in Table 1. Among these introduced detection methods, impedance-based droplet detection can achieve decently high throughput; however, it is done with limited specificity when interrogating the cellular phenotypes where the level of response difference is subtle. Scattering-based detection methods (Raman spectroscopy) can provide high-resolution characterization of in-droplet content, but they are bottlenecked with throughput; fluorescence-based droplet detection methods can balance both aspects, but they have additional concerns in the labeling process and the complexity of laser detection systems. Overall, we think fluorescent-based droplet detection has the greatest potential to be tailored against various application needs and achieve droplet interrogation in a high throughput manner. In the case where the goal is to detect the presence of the target cells/particles, an impedance-based system might be ideal for its comparably easy and cost-effective nature. As to the system cost, the Raman and visible detection system costs are the highest, as the high-speed camera or Raman spectroscopy are needed for building those systems. The fluorescent detection setup required PMT and a laser, which are still expensive but are relatively much cheaper than a high-speed camera or Raman microscope. The impedance detection only requires an impedance analyzer and, hence, is considered a cheaper option. Absorbance methods require only LED and photodiode, thus becoming the cheapest setup.

For droplet sorting methods, the accuracy of manipulation, biocompatibility, and throughput are three critical aspects when evaluating the applicability in the context of droplet screening applications. We have summarized the performance metrics for the aforementioned droplet sorting methods, including their achievable throughput, sorting accuracy, and reported applications in Table 2. Among introduced methods, passive methods are not well suited because almost all ideally processed droplets are with the same physical characteristics; magnetic-based sorting methods would require the pre-labeling of the target droplet, thus limiting their applications; thermal-based and pneumatic-based droplet sorting are both relying on the mechanical changes in the system and, therefore, have slow respond/recover times, which will induce false sorting when throughput exceeds few droplets per second. Acoustic-based droplet sorting has been well accepted as a practical method to be used in droplet screening applications due to its high throughput and high accuracy manner. The heating issue and device design would require some additional expertise and caution, as this might hinder the overall performance, both in terms of biocompatibility and energy efficiency. Lastly, dielectrophoretic-based droplet manipulation has been developed to a point where it has become very easy to fabricate and use cost-effectively, accurately, and with the highest throughput reported. With the most recently developed technologies, dielectrophoretic sorting can minimize its input power and further reduce the potential damage to biological samples. We consider it to be the most practical and robust system to be used in such applications.

Although various types of technologies have been developed to facilitate the sorting/detection functions in droplet microfluidics, only a few commercial systems have been successfully integrated (for example, On-chip Emulsion Sorting System). Several factors might contribute to this: first, biological applications are vastly different and, therefore, a generic applicable system is quite hard to achieve, so integrated system commercialization is quite challenging due to a limited market; second, most detection methods require high maintenance both in terms of calibration and operation; third, although microfluidic chips are miniaturized, the associated equipment setups would typically require huge footage, which might restrict the mobile and remote applications. Additional efforts regarding these aspects would be needed to further improve the detection and sorting system to enhance the availability of droplet microfluidic systems in commercial formats.

## 5. Conclusions

In this review, we summarize emerging technologies for droplet detection and sorting systems. According to their detection methods, we categorized and discussed impedance-based, fluorescent emission-based, scattering-based, and absorbance-based droplet detection methods. We also reviewed passive, dielectrophoretic, acoustophoretic, magnetophoretic, pneumatic, and thermal-based methods to compare the advantages, disadvantages, and their applications. We hope that this review can help researchers with various interests to lay the ground for recent progress on droplet detection and sorting systems, to identify the most suitable methods, and utilize such powerful tools to conduct their research.

## Figures and Tables

**Table 1:** Summary of all reviewed detection methods and their critical performance factors.

Detection Methods		Throughput	System Cost	Specificity	Applications	Ref
Impedance		0.1~3.3 kHz	Low	Medium	Empty droplet, viable/non-viable cells	[69,190]
Fluorescent		10~30 kHz	Medium	High	Yeast, filamentous fungi, microalgae, bacteria, direct evolution (cell-free) and particle detection (cell-free)	[79,170,191,192,193,194]
Absorbance		1~2.1 kHz	Lowest	Low	Chemical identification	[122,123]
Scattering	Raman	10~42 Hz	High	High	Microalgal cells, chemical polymerization and lipid production	[195,196,197]
	Visible	0.25~3 kHz	High	Low	Droplet PCR, single-cell screening	[124,125,126,127]

**Table 2:** Summary of all reviewed sorting methods and their critical performance factors.

Sorting Methods	Types	Throughput	Accuracy	Applications	Ref
Pneumatic	Single Layer	1~250 Hz	>99%	Single cell droplet, protein analysis	[136,137]
	Multi-Layer	1~50 Hz	>98%		[138,139]
Dielectrophoretic	AC	Up to 30 kHz	>99%	Single-cell assays, direct evolution (cell-free)	[79,142,198,199]
	DC	100 Hz~1.1 kHz	92 ± 5.4%		[152,200,201,202]
Acoustophoretic	BAW	1~5 Hz	-	Empty droplet, drug toxicity test, particle sorting (cell-free), on-demand droplet generation	[169,170]
	SSAW	222~1 kHz	99.2%		[47,179]
	TSAW	1~1 kHz	90~100%		[118,181,182,183]
Magnetophoretic	Perm Mag	100 Hz	95%	Empty droplet, cell viability assay, Single-cell analysis	[51,184]
	Electro Mag	0.5~50 Hz	58~90%		[185]
Thermal	Laser Focus	<1 Hz	100%	Empty droplet	[52,187,188]

## Data Availability

Not applicable.
